# Clarifying Recent Adaptive Diversification of the *Chrysanthemum-*Group on the Basis of an Updated Multilocus Phylogeny of Subtribe Artemisiinae (Asteraceae: Anthemideae)

**DOI:** 10.3389/fpls.2021.648026

**Published:** 2021-05-26

**Authors:** Chu-Ze Shen, Chu-Jie Zhang, Jie Chen, Yan-Ping Guo

**Affiliations:** ^1^MOE Key Laboratory for Biodiversity Science and Ecological Engineering, College of Life Sciences, Beijing Normal University, Beijing, China; ^2^School of Life Sciences, Peking University, Beijing, China; ^3^Key Laboratory of Plant Hormones and Development Regulation of Chongqing, School of Life Sciences, Chongqing University, Chongqing, China; ^4^Center of Plant Functional Genomics, Institute of Advanced Interdisciplinary Studies, Chongqing University, Chongqing, China

**Keywords:** *Chrysanthemum*, *Ajania*, coalescence, phylogeny, niche differentiation

## Abstract

Understanding the roles played by geography and ecology in driving species diversification and in the maintenance of species cohesion is the central objective of evolutionary and ecological studies. The multi-phased orogenesis of Qinghai-Tibetan Plateau (QTP) and global climate changes over late-Miocene has profoundly influenced the environments and evolution of organisms in this region and the vast areas of Asia. In this study, we investigate the lineage diversification of *Chrysanthemum*-group in subtribe Artemisiinae (tribe Anthemideae, Asteraceae) likely under the effects of climate changes during this period. Using DNA sequences of seven low-copy nuclear loci and nrITS and the coalescent analytical methods, a time-calibrated phylogeny of subtribe Artemisiinae was reconstructed with emphasis on *Chrysanthemum*-group. The monophyletic *Chrysanthemum-*group was well resolved into two major clades corresponding to *Chrysanthemum* and *Ajania*, two genera which can be well identified by capitulum morphology but have been intermingled in previous plastid and ITS trees. Within *Chrysanthemum*, a later divergence between *Ch. indicum*-complex and *Ch. zawadskii*-complex can be recognized. The time frames of these sequential divergences coincide with the late Cenozoic uplift of the Northern QTP and the concomitant climatic heterogeneity between eastern and inland Asia. Reconstruction of historical biogeography suggested the origin of *Chrysanthemum*-group in Central Asia, followed by eastward migration of *Chrysanthemum* and *in situ* diversification of *Ajania*. Within *Chrysanthemum*, *Ch. indicum*-complex and *Ch. zawadskii*-complex exhibited contemporary distributional division, the former in more southern and the latter in more northern regions. The geographic structure of the three lineages in *Chrysanthemum*-group have been associated with the niche differentiation, and environmental heterogenization in Asia interior.

## Introduction

Speciation is usually associated with morphological innovation or modification that occurs for internal molecular and/or external environmental reasons. Geographic isolation and ecological segregation are both important external forces for speciation (Coyne and Orr, 2004). Understanding the relative roles of geographic and ecological factors in the increase of species diversity and maintenance of species coherence is one of the central tasks of evolutionary biology (Wiens and Graham, 2005; Rundle and Nosil, 2010, 2012; Anacker and Strauss, 2014; Ebersbach et al., 2018).

In East Asia, the process of species diversification has been greatly influenced by the topographic activities of the Northern Qinghai-Tibet Plateau (QTP) and the aridification in Asian interior (Zheng et al., 2000; An et al., 2001; Guo et al., 2004; Miao et al., 2012; Ge et al., 2013; Li et al., 2015; Shi et al., 2015; Spicer, 2017; Shi et al., 2019; Ding et al., 2020). To what extent have ecogeographical gradients and diverse macrohabitats in this region promoted speciation (He et al., 2010; Qin et al., 2013; Yan et al., 2013)? To gain insight into this issue, comparative phylogeographic analyses of closely related species are required as such studies may help us trace trajectories of lineage splitting and reuniting (if these events happened) and infer evolutionary forces behind rapid speciation (Anacker and Strauss, 2014; Ortiz-Rodriguez et al., 2018; Wang Z. M. et al., 2019; Knope et al., 2020).

*Chrysanthemum*-group, one of the youngest branches of the largest angiosperm family, Asteraceae, is a proper system for studying the aforementioned problem. This group belongs to subtribe Artemisiinae, tribe Anthemideae of Asteraceae (Bremer and Humphries, 1993; Watson et al., 2002; Kondo et al., 2003; Sanz et al., 2008; Masuda et al., 2009). According to their cladistic analysis of morphological characteristics, Bremer and Humphries (1993) defined 18 genera in subtribe Artemisiinae. This circumscription has been widely accepted with the later incorporation of *Hippolytia*, *Opisthopappus*, and *Tanacetum tatsienense*, which formerly belonged to subtribe Tanacetinae, as well as *Leucanthemella* and *Nipponanthemum*, which were formerly in subtribe Leucantheminae. Despite of its uncertain circumscription, the subtribe Artemisiinae is mainly composed of species belonging to two groups, *Chrysanthemum*-group and *Artemisia-*group (Bremer and Humphries, 1993; Oberprieler et al., 2007; Sanz et al., 2008; Oberprieler et al., 2009; Sonboli et al., 2012).

*Chrysanthemum*-group was historically recognized mainly by solitary flower heads or corymbose synflorescences (in contrast to *Artemisia-*group with paniculate synflorescences), radiate capitula (*Chrysanthemum*, *Arctanthemum*, and *Brachanthemum*) or disciform capitula (*Ajania* and *Phaeostigma*), and echinate *Anthemis*-type pollen grains (except *Phaeostigma* with microechinate *Artemisia*-type) (Bremer and Humphries, 1993; Sanz et al., 2008; Pellicer et al., 2010). However, the circumscription and monophyly of the two groups have remained questionable, and the generic relationships within each group have been rather controversial (Bremer and Humphries, 1993; Torrell et al., 1999; Oberprieler et al., 2007; Zhao et al., 2010a, b). These problems are probably due to ongoing speciation, including recent divergence and secondary contacts of lineages (Masuda et al., 2009; Miao et al., 2011; Liu et al., 2012b; Li et al., 2014; Chen et al., 2020).

Within *Chrysanthemum-*group, there are two major genera, *Chrysanthemum* and *Ajania*, both mainly distributed in East Asia and each consisting of 30–35 species (Shih and Fu, 1983; Oberprieler et al., 2006). Poljakov (1955) speculated that *Ajania* was closely related to *Artemisia*, but Tzvelev (1961) considered *Ajania* and *Chrysanthemum* to be sister lineages derived from a most recent common ancestor that had radiate capitula (Muldashev, 1983; Bremer and Humphries, 1993). *Ajania* was even once treated as a section under *Chrysanthemum* (Kitamura, 1978; Kishimoto et al., 2003; Ohashi and Yonekura, 2004). Considering their distinct capitulum morphologies, we have postulated that these two genera must have experienced adaptive divergence associated with differential environmental conditions (Chen et al., 2020). Our recent developmental genetic study revealed that a disciform capitulum may have evolved from a radiated type owing to the dysfunction of a key ray-flower regulator, CYCLOIDEA2g (Chen et al., 2018; Shen et al., 2021). To date, however, no molecular phylogeny has resolved *Chrysanthemum* and *Ajania* each as monophyletic (Kondo et al., 2003; Masuda et al., 2009; Zhao et al., 2010a, b; Liu et al., 2012b; Huang et al., 2017). In another aspect, the small genus *Phaeostigma* (established by Muldashev in 1981) was once described under *Ajania* (Muldashev, 1981), but was clarified by our previous analysis as a monophyletic group that is probably closer to *Artemisia* than to *Ajania* despite its corymbose synflorescence similar to that of the latter (Huang et al., 2017). *Brachanthemum*, which was supposed to be closely related to *Chrysanthemum* due to its thin-walled and pappus-lacking achenes (Bremer and Humphries, 1993), was suggested by molecular phylogeny to be closer to *Nipponanthemum*, *Leucanthemella*, or *Kaschgaria* than to *Chrysanthemum* (Vallès et al., 2003; Sanz et al., 2008; Masuda et al., 2009; Zhao et al., 2010a). As to the monotypic genus *Arctanthemum*, it has been incorporated into *Chrysanthemum* in the floristic work of China (Shih and Fu, 1983) thus the name *Chrysanthemum arcticum* was used instead of *Arctanthemum arcticum*.

In terms of geography, *Chrysanthemum*-group is widely distributed in Middle to East Asia, with a few members extending to Central Europe and North America (Shih and Fu, 1983). Two major genera, *Ajania* and *Chrysanthemum*, cover most of its full range of distribution, with *Ajania* in China (from northwestern to northeastern and southwestern parts), Korea, Japan, and the Far East, and *Chrysanthemum* in the more eastern part of China, Korea, Japan, and Russia (Shih and Fu, 1983; Zhao et al., 2009). Within *Chrysanthemum*, there are two species complexes corresponding to morphological characteristics and weakly supported by the chloroplast phylogeny—*Ch. zawadskii-*complex, with relatively larger flower heads and white to purple ray flowers, and *Ch. indicum-*complex, with smaller flower heads and white or yellow rays (Shimizu, 1961; Lee, 1969; Liu et al., 2012b; Li et al., 2013, Li et al., 2014; Kim et al., 2014; Meng et al., 2020). Considering geographic distribution, *Ch. zawadskii*-complex is distributed mainly in the northern region of East Asia, while *Ch. indicum*-complex is relatively in the more southeastern part (Shih and Fu, 1983; Shishkin and Bobrov, 1995; Zhao et al., 2009).

Untangling recent speciation events usually requires a reliable phylogenetic framework. To date, all deep phylogenetic relationships within subtribe Artemisiinae inferred from plastic and nrITS markers have provided limited information (Watson et al., 2000, 2002; Sanz et al., 2008; Pellicer et al., 2010; Zhao et al., 2010a; Liu et al., 2012b). Here, we utilized multilocus nuclear DNA sequences and a coalescent analytical method to update the tree of this subtribe with emphasis on *Chrysanthemum*-group. Subsequently, we estimated the optimal ancestral distribution and biogeographical history of the major clades in this subtribe. Furthermore, we conducted ecological niche modeling and niche overlap tests to verify the ecological differentiation of lineages within *Chrysanthemum*-group and to see whether patterns of geographic distribution were linked to environmental conditions. With all these analytical results, we attempted to resolve possible rapid species divergence under macrohabitat differentiation in interior East Asia.

## Materials and Methods

### Taxon Sampling

Taxon sampling for this study covered all major branches of subtribe Artemisiinae. In total, 101 accessions of 96 species were sampled. Of the 96 species, 53 were of *Chrysanthemum*-group, including 30 out of approximately 35 *Chrysanthemum* species, 20 out of approximately 30 *Ajania* species, one species of the monotypic genus *Elachanthemum*, and both the two species of *Opisthopappus*. To clarify generic relationships within subtribe Artemisiinae, 23 species covering all four commonly accepted subgenera of *Artemisia* and its close allies were included in this study. Also sampled were *Phaeostigma, Brachanthemum*, and *Kaschgaria*, which were of uncertain phylogenetic positions in Artemisiinae, as well as *Hippolytia*, *Nipponanthemum*, *Leucanthemella*, and *Tanacetum tatsienense*, which were later placed in Artemisiinae. Six species of four subtribes that belong to the Eurasian-Mediterranean clade of tribe Anthemideae (Oberprieler et al., 2007) were also sampled and used as outgroups. The detailed sampling information is listed in Supplementary Table 1.

Leaf samples were mostly collected from the wild, while some were obtained from seedlings geminated from seeds from international seed banks^1^. Voucher specimens were deposited in the herbarium of Peking University, and living seedlings were grown in the greenhouse of Peking University.

### DNA Extraction and Gene Isolation

Genomic DNA was extracted from silica gel-dried or fresh leaves with a Plant Genome Extraction Kit (Tiangen Biotech, China) following the manufacturer’s protocol. For better phylogenetic resolution, we utilized nrITS and seven low-copy nuclear genes, *AGO1* (*ARGONAUTE 1*; Zhang et al., 2015), *BRC1* (*BRANCHED1*; Zhou et al., 2012; Wang M. et al., 2019), *CDS* (chrysanthemyl diphosphate synthase gene; Rivera et al., 2001; Liu et al., 2012a), *F3’H* (flavonoid3′-hydroxylase gene; Zhao et al., 2013), *LFY* (*LEAFY*; Ma et al., 2016), *NAM* (*No Apical Meristem*; Sha et al., 2017) and *UEP1* (gene of ubiquitin extension protein; Annadana et al., 2002). Polymorphic regions mostly covering introns and 5′UTRs of five of the six genes were amplified using conserved primer pairs that were developed according to sequences acquired from GenBank (Table 1). To isolate *AGO1* orthologs from the species of interest, we downloaded the *AGO1* sequence of *Helianthus tuberosus* and then ran a local BLAST in the genome data of *Ch. nankingense* (Song et al., 2018). The primer pair was designed based on the orthologous sequence of *Ch. nankingense* (Table 1). All PCR products were ligated into pGEM-T vectors (Promega, United States) and cloned. At least 4–6 positive clones were randomly taken for sequencing. DNA sequences obtained by this study are deposited in GenBank with accession numbers MW344433–MW344631 (*AGO1*), MW195142–MW195312 (*BRC1*), MW543604–MW543703 (*CDS*), MW543450–MW543603 (*F3’H*), MW011041–MW011206 (*LFY*), MW195313–MW195497 (*NAM*), MW344310–MW344432 (*UEP1*), and MW545598–MW545801 (nrITS).

**TABLE 1:** Primers for the amplification of nuclear genes in this study.

**Locus**	**Primer name**	**Primer sequence**	**Developed by**	**Accession No. of the ref. sequence**
AGO1	AGO1_CKR	AAAAGGGAGAGGCCCAGCCGTAT	This study	MG710521.1
	AGO1_utrF	AGCCACAGCAACAGGGTGGCTAT		
BRC1	BRC1aR	AATCTCAAACACCCCTTGACACT	This study	JX870411.1
	BRC1aF	CCATCATTTTCCTCATTCCGCCT		
CDS	CDS II	CTTSTMCWTGATGACATRATGGA	Liu et al., 2012a	
	CDS Vb	TGCATTCTTCAATATCTGTTCCMGT		
	CDS IIa	ATGRATGSCTCBCAYACACG		
	CDS Va	CRAAAGTGTCGAGATAATCATT		
F3′H	F3′H_int2F	GCTGATATTGAAGGTGGGAAGCT	This study	MF663713.1 and AB523844.1
	F3′H_int2R	AATGAGTTCGGCTATTGCCCATT		
ITS	ITS1	AGAAATCGTAACAAGGTTTCCGTAGG	Li et al., 2014	
	ITS4	TCCTCCGCTTATTGATATGC		
LFY	LFY_int2F	TGTCGTGAGTTCTTGGTCCAAGT	This study	KF151334.1
	LFY_int2R	TGAGTTTGGTTGGGACATACCAT		
NAM	NAM_int2R	CTCTTCTTGAACACACGTGAGAT	This study	KX722453.1
	NAM_int2F	TGGGTTATGCATGAATATCGTCTT		
UEP1	UEP1-R	AGATCATCAATTGGGTGTCCCAT	This study	EU862325
	UEP1-F	GCCCACACCATATAAAGCCGATT		

### Phylogenetic Analysis

To guarantee that the fragments we sequenced for each gene were orthologous, reciprocal BLAST of the obtained sequences was conducted (Wall and Deluca, 2007). Then, alignment was performed using the online toolkit MAFFT v.7^2^ with default parameters followed by manual checking in MEGA 7 (Kumar et al., 2016). Highly polymorphic positions including multiple gaps that made alignment ambiguous were removed from the alignment. For each nrITS sequence, ITS1 and ITS2 were tandemly linked with 5.8S-rRNA excluded. Subsequently, we used DAMBE v. 6.4.101 (Xia, 2017) to merge identical sequences within each species. The aligned data matrix of each of six low-copy genes was subjected to a prior Bayesian tree with which we found no evidence showing paralogous copies (Supplementary Figure 1) [As *CDS* gene was previously proved as single-copy (Rivera et al., 2001; Liu et al., 2012a), it was not shown in Supplementary Figure 1]. To construct the Bayesian tree, the best-fit evolutionary model of each dataset was selected using jModeltest 2.1.7 (integrated in online bioinformatic platform CIPRES^3^) according to the Bayesian informatic criterion (BIC) values (Posada, 2008; Table 2).

**TABLE 2:** Sequence information of the present phylogenetic reconstruction.

**Dataset**	**Locus**	**Num. of taxa**	**Num. of sequences**	**Aligned length**	**Variable sites**	**Parsimony informative sites**	**Model selected by BIC**
Subtribe Artemisiinae	AGO1	101	198	805	459 (57.0%)	291 (36.1%)	TN + G
	BRC1	101	170	1229	790 (64.3%)	534 (43.4%)	TN + G + I
	F3’H	101	153	289	189 (65.4%)	154 (53.3%)	HKY + G + I
	ITS	101	101	403	191 (47.4%)	124 (30.7%)	TN + G
	LFY	101	164	1464	937 (64.0%)	664 (45.4%)	HKY + G + I
	NAM	101	181	751	543 (72.3%)	369 (49.1%)	GTR + G
*Chrysanthemum*-group	AGO1	57	120	808	271 (33.5%)	126 (15.6%)	TN + G
	BRC1	57	103	1210	437 (36.1%)	219 (18.1%)	TN + G + I
	CDS	57	101	356	157 (44.1%)	69 (19.4%)	HKY + G + I
	F3’H	57	83	557	238 (42.7%)	159 (28.5%)	HKY + G + I
	ITS	57	57	403	81 (20%)	30 (7.4%)	TN + G
	LFY	57	98	1290	600 (46.5%)	369 (28.5%)	HKY + G
	NAM	57	107	751	361 (48.1%)	183 (24.4%)	GTR + G
	UEP1	57	124	503	290 (57.6%)	164 (32.5%)	HKY + G

The tree of the whole subtribe Artemisiinae was constructed based on six of the eight loci, as the targeted fragments of *CDS* and *UEP1* could not be amplified from some *Artemisia* species and the outgroups. The phylogenetic reconstruction of subtribe Artemisiinae followed a coalescent strategy using ^∗^BEAST embedded in BEAST v. 1.8.4 by selecting the Yule tree prior and the Piecewise linear and constant root of population size model (Drummond et al., 2012a, b; Liu et al., 2015; Edwards et al., 2016). The divergence times of major nodes were estimated under uncorrelated relaxed clock by setting two secondary calibration points with normal distributions: The first was the divergence time of the Eurasian-Mediterranean clade and the Asian-southern African clade (17.3 Ma ± 1 SD), and the second was the age of the crown clade of Artemisiinae (9.7 Ma ± 1 SD) according to Oberprieler (2005). To test the influence of different time-point settings on molecular dating, the secondary calibration point was set according to Tomasello et al. (2015) (the divergence time of Artemisiinae and Santolininae as 18 Ma ± 1 SD; or the divergence time of Artemisiinae-Santolininae-Glebionidinae and other Eurasian lineages as 22.5 Ma ± 1 SD). Moreover, the fossil records of *Artemisia*-like pollens were also considered for calibration. We set tmrca prior at 13 Ma ± 1 SD for the crown lineage of Artemisiinae (the stem node of lineages sharing *Artemisia*-type pollens, e.g., *Artemisia*, *Elachanthemum*, and *Phaeostigma*) according to the records of the commonly occurred *Artemisia*-like pollen fossils (Wang and Zhang, 1990; Ma et al., 2005). Trees were sampled every 10000 generations for a total of 400 million generations. We checked for topological convergence and adequate ESS (>200) using Tracer v.1.7.1 (Rambaut et al., 2018). The consensus tree was exported using TreeAnnotator v. 1.8.4, discarding the first 30% of trees as burn-in.

Then, all eight loci were applied to construct a tree of *Chrysanthemum*-group to better resolve relationships within this particular group following the same analytical method specified above.

### Estimation of Diversification Dynamics

Subsequently, 5000 trees from the ^∗^BEAST analysis were resampled to construct a lineage-through-time (LTT) plot using the R package *ape* v. 5.0 (Paradis and Schliep, 2019). Then, 1000 random trees were simulated under birth-death (BD) and pure-birth (PB) models using the R package *geiger* to avoid errors due to incomplete sampling (Pennell et al., 2014). These simulations were used to establish 95% confidence intervals on the LTTs for each model for comparison with the empirical dataset. The γ statistic for the optimal ^∗^BEAST phylogeny was calculated using *ape* v. 5.0. Speciation rates were compared with the null hypothesis that a clade diversified at a constant rate using 2^∗^[1-pnorm(abs(gammaStat(tree)))] provided by the R package *ape* v. 5.0 for a two-tailed test (Paradis and Schliep, 2019). A significantly positive or negative γ value meant an accelerated/decelerated rate of speciation toward the present.

### Reconstruction of Ancestral Distributions and Character States

To infer the biogeographic history of lineages in subtribe Artemisiinae, dispersal and vicariance analysis (s-DIVA), Bayesian binary MCMC analysis (BBM) and dispersal-extinction-cladogenesis (DEC, s-DEC) were conducted to estimate the optimized geographical distributions of internal nodes using RASP 4.0 (Yu et al., 2015). The distribution information of all samples was obtained from floristic works (Shih and Fu, 1983; Shishkin and Bobrov, 1995; Zhao et al., 2009) and herbarium specimens. Based on this information and major biogeographic boundaries, eight geographic units were defined: (A) Europe and the Mediterranean coast; (B) Middle Asia; (C) Central Asia; (D) the QTP region; (E) the southern part of East Asia; (F) the northern part of East Asia; (G) Korea-Japan; and (H) the pan-Arctic region (Spencer and Robinson, 1968; Xie et al., 2002; Cowan, 2007; Lu et al., 2018). The ranges of geographic units are as shown in Supplementary Figure 2. The current distribution of each species is marked by a particular color before the species name. For s-DIVA, s-DEC and DEC, 10000 trees that were resampled from the ^∗^BEAST phylogenies after burn-in and the consensus tree were loaded into RASP to estimate the likelihoods of ancestral states at each internal node of the consensus tree. BBM was run with F81 state frequencies using gamma variation for 1,000,000 iterations. The reconstructed state was sampled every 1000 generations, and the first 10% was discarded as burn-in.

To estimate the ancestral states of flowerhead architectures, character states were mapped onto terminal branches of the consensus tree resulting from the ^∗^BEAST analysis, and then searches for optimized states at the internal nodes were run with the likelihood model in Mesquite v. 3.5.2 and with the continuous-time Markov model using the *phytools* v. 0.7-70 package in R (Maddison and Maddison, 2009; Revell, 2012).

### Ecological Niche Modeling, Lineage Distribution Models and Niche Overlap Tests

To analyze niche differentiation and to understand how the distributions of three major clades of *Chrysanthemum*-group changed during the last glacial maximum (LGM), niche modeling was conducted under recent climatic scenarios and LGM scenarios from the CCSM4 model (current climate at a 30 arcsec resolution and LGM at a 2.5 arcminute resolution). Ecological niche data including temperature and precipitation (19 bioclimatic variables) data that were drawn as climate layers were downloaded from WorldClim 1.4^4^. Coordinate information was collected from occurrence records in the Global Biodiversity Information Facility (GBIF^5^) and from herbarium specimen information acquired from NSII^6^. For every two occurrences, only one was kept if less than 1 km in distance. All of the coordinates used for inferring lineage distribution modes are listed in Supplementary Table 2. In total, the current environmental data of 1249 occurrence records, including 529 of *Ajania*, 529 of *Ch. indicum*-complex and 191 of *Ch. zawadskii*-complex were sampled. To avoid collinearity, Pearson pairwise correlation analysis of environmental factors was conducted. For each pair, one factor with a correlation value (| R|) higher than 0.75 was eliminated, and the result was visualized in SPSS 22 and Heml (Deng et al., 2014). We used lineage geographic distribution spots to estimate the environmental space of each of the three main subclades of *Chrysanthemum*-group with the PCA-env approach in SPSS 22 and R (R Core Team, 2013). Species distribution models (SDMs) were built under the maximum-entropy method implemented in MAXENT 3.3 following Papeş and Gaubert (2007) for parameter settings (25% of occurrence records were used as testing data) (Papeş and Gaubert, 2007; Phillips and Dudík, 2008). The area under the receiver operating characteristic curve (AUC) was used to evaluate the prediction performance of the models (Phillips et al., 2006). AUC values > 0.75 indicate good predictions, whereas values < 0.5 suggest poor predictions (not better than random). We also used the true skill statistics (TSS) to evaluate the accuracy of the resulting distribution models (Liu et al., 2005; Allouche et al., 2006), where TSS values ranging from 0.4 to 0.8 are indicative of good model performance (Landis and Koch, 1977; Fielding and Bell, 1997). The 10-percentile training presence threshold was used to generated binary map. Subsequently 10000 random geographic points were extracted to calculated TSS values for each niche model in R. Moreover, DIVA-GIS v. 7.5 was applied to model potential distributions under the current scenario to ensure the reliability of SDMs (Hijmans et al., 2001). To quantify climatic niche overlap, Schoener’s *D*-index (Schoener, 1970) and the modified Hellinger distance *I*-index (Van der Vaart, 2000) were calculated in R. Subsequently, niche equivalency and similarity tests (Evans et al., 2009; Warren et al., 2008) were performed between subclades using 50 permutations in the R packages *phyloclim* v. 0.9.5 and *ENMtools* v. 1.0.2 (Warren et al., 2010; Heibl and Calenge, 2013). To analyze pairwise differentiation in detail on every explanatory bioclimatic variable resulting from analysis by PCA-env, the profiling niche occupancy (PNO) of each climatic factor was computed, and then the *D*-index and the modified *I*-index were calculated using the *phyloclim* v. 0.9.5 package in R (Heibl and Calenge, 2013).

## Results

### Sequence Characteristics

Despite repeated amplifications, we failed to isolate the target *CDS* fragment from some species, e.g., *Artemisia frigida* and *Seriphidium finitum*, and we failed to isolate its complete open reading frame in *Nipponanthemum nipponicum*, *Hippolytia* spp. We were not able to obtain *UEP1* from *Stilpnolepis centiflora* and the outgroup taxa. Therefore, the analyses were conducted separately with two datasets, with one excluding *CDS* and *UEP1* for all the sampled taxa of subtribe Artemisiinae and the other including all eight loci but limited to *Chrysanthemum*-group. For each of the datasets, the number of species/taxa included, the number of haplotypes obtained at each locus and other information related to phylogenetic analyses are given in Table 2.

### Phylogeny of Subtribe Artemisiinae and the Estimation of Diversification Dynamics

**FIGURE 1:**
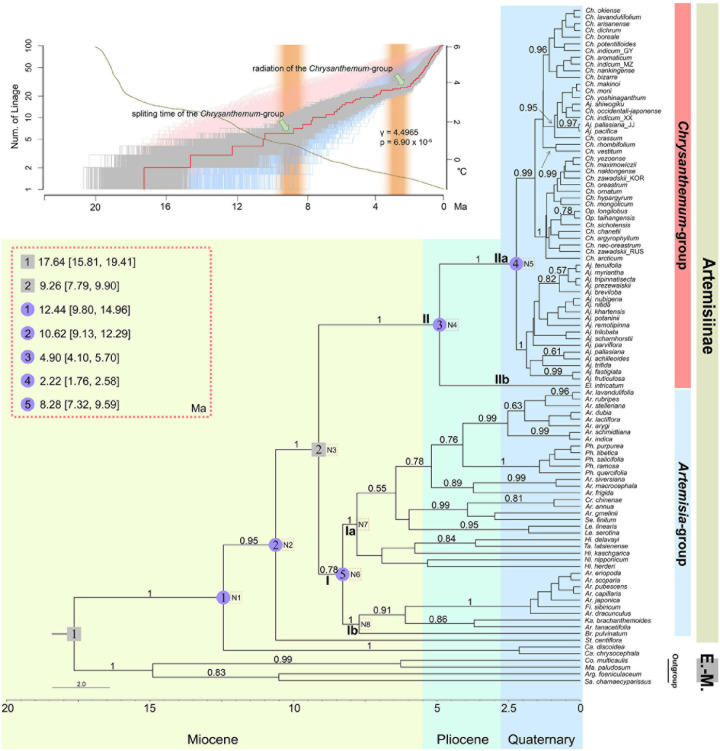
Coalescent species tree inferred from six nuclear gene sequences and the lineage through time (LTT) plot of subtribe Artemisiinae. The tree was constructed in *BEAST and rooted by four Eurasian-Mediterranean (E.-M.) species of tribe Anthemideae. Bayesian posterior probabilities (>0.5) are indicated above branches. Two secondary calibration time points used for estimating divergence times are marked with gray squares. Five major clades are highlighted by blue circled numbers, and their estimated divergence times are noted in the upper-left box. Blue, green, and yellow blocks indicate different geologic ages. The dotted square with N1–N8 inside indicates the nodes analyzed for biogeographic history, as shown in Table 3. Abbreviations of genus names: *Ch.*, *Chrysanthemum*; *Op*., *Opisthopappus*; *Aj*., *Ajania*; *El*., *Elachanthemum*; *Ar*., *Artemisia*; *Ph*., *Phaeostigma*; *Cr*., *Crossostephium*; *Se*., *Seriphidium*; *Le*., *Leucanthemella*; *Ni*., *Nipponanthemum*; *Hi*., *Hippolytia*; *Ta*., *Tanacetum*; *Fi*., *Filifolium*; *Ka*., *Kaschgaria*; *Br*., *Brachanthemum*; *St*., *Stilpnolepis*; *Ca*., *Cancrinia*; *Co*., *Coleostephus*; *Ma*., *Mauranthemum*; *Sa*., *Santolina*; *Arg*., *Argyranthemum*. The LTT plot using the uncorrected relaxed clock model in *BEAST is shown in the upper-left corner in gray, where the red line indicates the LTT plot of the maximum credibility tree for the *BEAST analysis. Lineages accumulated over time under the pure-birth model are marked in blue, and those under birth-death are marked in pink. The thin dotted line represents the decrease in the environmental temperature during the time frame of diversification of subtribe Artemisiinae, referring to Hansen et al. (2013). Statistic γ takes on positive values when there are accelerated speciation rates toward the present; *p* indicates the significance value (< 0.001). Two brown columns mark the periods of uplift of the Northern QTP during the late Miocene.

The coalescent species tree of Artemisiinae overall was inferred from six of the eight markers (Figure 1). Rooted by outgroup species from the Eurasian-Mediterranean clade of tribe Anthemideae (Oberprieler et al., 2007), the tree showed subtribe Artemisiinae as monophyletic. Except for the basal monotypic genus *Stilpnolepis*, two main clades, Clades I and II, were found (Figure 1: posterior probabilities PP = 0.78 and 1, respectively). These two clades roughly corresponded to but differed slightly in circumscriptions from the *Artemisia-*group and *Chrysanthemum*-group traditionally defined by morphological characteristics. The species of *Artemisia* and its allies, e.g., *Filifolium* and *Crossostephium*, were found in Clade I. However, the broadly defined *Artemisia* was not supported as monophyletic. Several genera with radiate capitula but uncertain phylogenetic positions, such as *Leucanthemella*, *Nipponanthemum*, *Hippolytia*, *Brachanthemum*, and *Tanacetum tatsienense*, fell into this clade. Two subclades, Ia and Ib, were further recognized with high posterior probability (PP = 1). Notably, the present data showed that species of *Phaeostigma* were clustered in subclade Ia, supporting the proposal that they were independent of *Ajania* but were a lineage within *Artemisia* (Figure 1). Subclade Ib contained species formerly described under *Artemisia* subgenus *Dracunculus*, as well as *Kaschgaria* and *Brachanthemum* (with radiate capitula) (Figure 1).

**TABLE 3:** Biogeographic history of subtribe Artemisiinae inferred by reconstructing ancestral distributions with BBM and s-DIVA.

**Node**	**s-DIVA**	**Event**	**BBM**	**Event**
N1	C (94.19%)		C (91.41%)	
N2	C (100%)		C (96.12%)	
N3	C (91.87%)	Vicariance	C (88.43%)	Dispersal
N4	C (63.17%)	Dispersal	C (95.24%)	
N5	C (65.52%)	Vicariance	C (91.92%)	
N6	C (85.95%)	Dispersal/Vicariance	C (59.15%)	Dispersal
N7	C (22.17%)	Dispersal/Vicariance	BC (40.86%)	Dispersal
N8	C (56.61%)	Dispersal	C (56.18%)	Dispersal

*N1-N8 are indicated in both Figure 1 and Supplementary Figure 2 near the corresponding nodes.*

**FIGURE 2:**
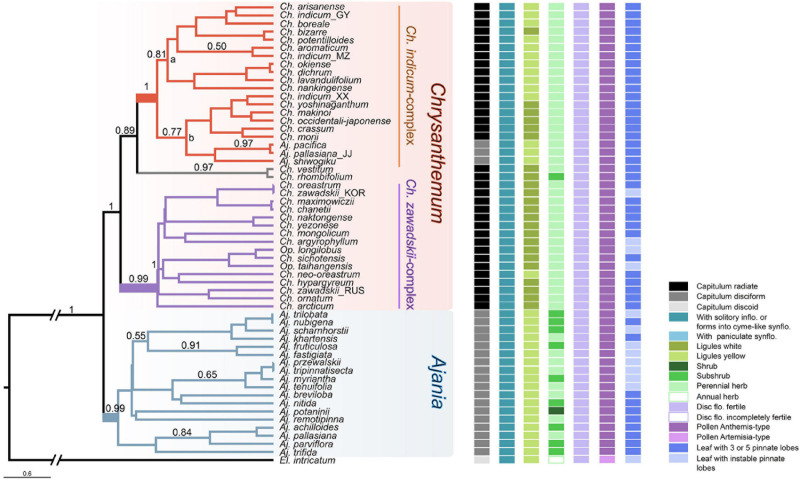
Coalescent tree of the *Chrysanthemum*-group inferred from eight nuclear gene sequences. Bayesian posterior probabilities higher than 0.5 are indicated on branches. States of important morphological characters (represented by small squares in different colors) are mapped at the right side of terminal nodes. In this tree, two subclades, *Ch. indicum*-complex and *Ch. zawadskii*-complex, can be recognized within *Chrysanthemum*. Naming of the two *Chrysanthemum* subclades follows Liu et al. (2012b).

Clade II included *Chrysanthemum* and *Ajania* and two small genera, *Elachanthemum* and *Opisthopappus*. Two sister branches, IIa and IIb were also found in Clade II: *Elachanthemum*, the only member of IIb, is different from *Chrysanthemum* and *Ajania* by having a discoid capitulum and an annual life form. Interestingly, *Opisthopappus*, which was formerly treated in subtribe Tanacetinae and then were incorporated into Artemisiinae (Bremer and Humphries, 1993; Oberprieler et al., 2009), was clustered in IIa; morphologically, this small genus is indeed similar to *Chrysanthemum* by having radiate capitulum and myxogenic achenes (Figures 1, 2).

The chronograms achieved using relaxed molecular clock analyses showed the time frames were largely similar among four different calibration settings (Supplementary Figure 3). According the chronogram inferred from setting two secondary calibration time points suggested that subtribe Artemisiinae began to diverge at approximately 10 Ma ago, and most of its major lineages diversified during the late Miocene, particularly densely at approximately 7-9 Ma ago (Figure 1). *Artemisia-*group split from *Chrysanthemum*-group at approximately 9 Ma ago and then diversified immediately, while the latter group diversified much later. Within *Chrysanthemum*-group, the monotypic genus *Elachanthemum* diverged much earlier during the mid-Pliocene (Figure 1, median age 4.90 Ma, 95% HPD 4.10-5.70 Ma), while other species seemed to have undergone evolutionary radiation since the boundary between the Tertiary and Quaternary (median age 2.22 Ma, 95% HPD 1.76-2.58 Ma). The LTT plots highlighted a significant speedup of diversification of subtribe Artemisiinae (γ = 4.50, *p* < 0.001), which was largely correlated with the cooling climate estimated by the δ^18^ O content (Figure 1; Hansen et al., 2013). The time frame of evolution of *Chrysanthemum*-group was in concordance with the intensive aridification of Middle to Central Asia that was probably due to late-Miocene to Pliocene geographic activity of the northern region of QTP and the global climatic changes (Figure 1).

### Internal Relationships Within the *Chrysanthemum*-Group

To better resolve relationships within *Chrysanthemum*-group, all eight nuclear gene sequences were used. As a result, two major genera, *Chrysanthemum* and *Ajania* were well separated into two clades (PP > 0.99) with a few exceptions (Figure 2), which provided important support for traditional taxonomy that treats them as independent genera mainly according to capitulum morphology. In *Chrysanthemum*, two Chinese endemic species with white ray flowers, *Ch. rhombifolium* and *Ch. vestitum*, formed a small clade sister to *Ch. indicum*-complex with yellow or white ray flowers (Figure 2). In the clade of *Ch. indicum*-complex, two branches, a and b, were recognized—species in ‘b’ basically all had island distributions, while species in ‘a’ were mostly distributed in mainland China (Figure 2). Another relatively large group is the so-called *Ch. zawadskii*-complex (purple-colored in the tree, PP = 0.99, Figure 2). This group is characterized by white to purple ray flowers with continuous color variation. Species of *Opisthopappus*, which were shown at the basal position of *Chrysanthemum-*group in the ITS and plastid DNA tree, fell into this clade. In contrast to *Chrysanthemum*, relationships within *Ajania* were still ambiguous, with most branches being poorly supported.

### Reconstructions of Ancestral Distributions of *Chrysanthemum*-Group

To explore the historical process of diversification of *Chrysanthemum*-group, the ancestral distribution state of each of the major nodes was estimated based on their recent distributions acquired from floristic works and specimen information. The Bayesian BBM analysis, s-DIVA analysis and DECs produced similar results regarding the ancestral area of Artemisiinae (Table 3 and Supplementary Table 3). Eight geographic units and optimal area reconstruction at each major node are summarized in Supplementary Figure 2. In RASP, both s-DIVA and BBM favored ancestral distributions of most of the major clades of Artemisiinae (N1-N8) in Central Asia (region C; >50%) (Table 3 and Supplementary Figure 2). Subsequent vicariance between Central Asia and other regions of Asia was reconstructed by s-DIVA for node N3 and N5, and dispersal from Central Asia to the QTP and eastern parts of Asia was reconstructed for nodes N4, N6-N8 and *Chrysanthemum*-group (Table 3). The time-effect curve reconstructed with BBM showed that the dispersal curve had extremely high peaks over the last 3 Ma (Supplementary Figure 2).

### SDMs and Niche Differentiation Among Lineages Within *Chrysanthemum*-Group

**FIGURE 3:**
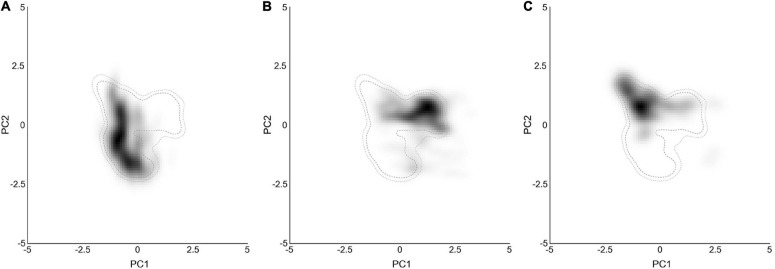
Ecological niches of the three analyzed subclades within the *Chrysanthemum*-group in environmental space produced by the principal component analysis method. The PCA results of **(A–C)** represent the niches of the lineages in the two main axes with the environmental conditions of sampled occurrences for *Ajania*, *Ch. indicum*-complex and *Ch. zawadskii*-complex, respectively. The gray-to-black shading shows the density of the occurrences by cell. The outer and inner dashed contour lines illustrate 100 and 95% of the available (background) environment, respectively.

To reduce multicollinearity, one factor of each pair of environmental factors was eliminated when the correlation coefficient of that pair was larger than 0.75. Thus, six factors were finally selected: isothermality (bio3), temperature seasonality (bio4), mean temperature of the wettest quarter (bio8), mean temperature of the driest quarter (bio9), annual precipitation (bio12) and the precipitation of the driest quarter (bio17) (Figure 3 and Supplementary Figure 4A). The variable loadings for PCA-env are shown in Table 4. The first two PCs explained 81.8% of the niche variation among subclades (51.1 and 30.7%, respectively). PC1 is dominated by the absolute temperature and precipitation variables (bio9, bio12, bio17) while PC2 by the two variables describing oscillations of temperature (bio3, bio4) (Table 4). The PCA-env assay suggested great variability in the environmental space inhabited by the different subclades within *Chrysanthemum*-group, especially between *Ajania* and *Ch. indicum*-complex (Figure 3 and Supplementary Figure 4).

**TABLE 4:** Loadings on the first two components for PCA-env analysis.

**Variables**	**PC1**	**PC2**
Isothermality (bio3)	–0.032	–0.951
Temperature sensonality (bio4)	–0.634	0.742
Mean temperature of wettest quarter (bio8)	0.569	0.519
Mean temperature of driest quarter (bio9)	0.901	–0.179
Annual precipitation (bio12)	0.917	0.07
Precipitation of driest quarter (bio17)	0.828	0.291
Cumulative variance	0.5106	0.8181

**FIGURE 4:**
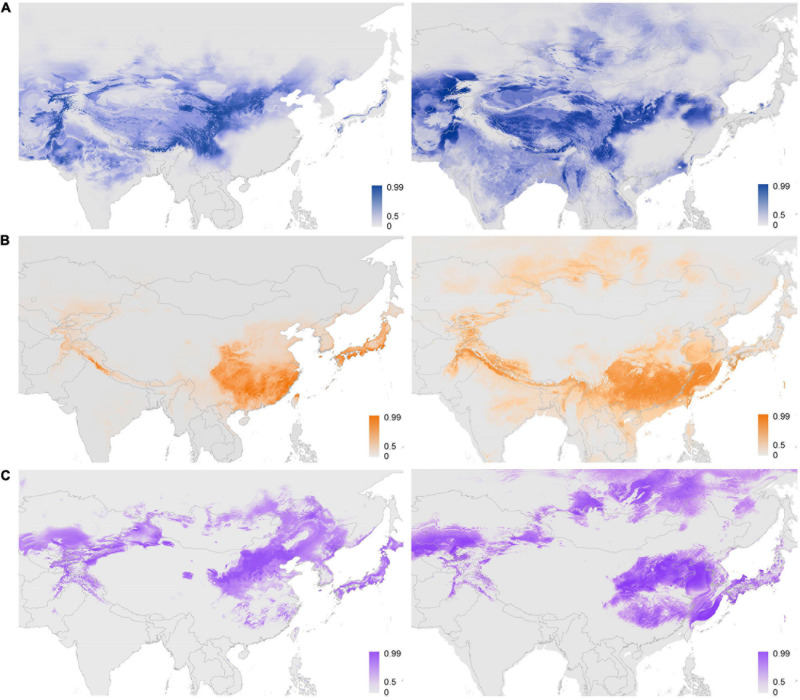
Potential distribution area of *Ajania* and two subclades of *Chrysanthemum* predicted by the maximum-entropy model. **(A–C)** Represent the predicted distributions of *Ajania*, *Ch. indicum*-complex and *Ch. zawadskii*-complex, respectively, during the current period (left) and during the Last Glacial Maximum (LGM) (right). Shadings in color show the probability of geographic occurrences. The prediction for the Korea–Japan distributed *Ajania* species is shown in Supplementary Figure 5.

Furthermore, the potential distribution of subclades within *Chrysanthemum*-group was predicted through MAXENT and DIVA-GIS. The results from both approaches were, in principle, similar, with the area predicted by DIVA-GIS being slightly restricted (Figure 4 and Supplementary Figures 5A-C). The AUC values for the replicate runs in MAXENT were 0.905 ± 0.003, 0.930 ± 0.003, and 0.907 ± 0.009 for *Ajania*, *Ch. indicum*-complex and *Ch. zawadskii*-complex, respectively. Establishing the threshold probability for niche models using TSS of each of three subclades resulted in 0.72 ± 0.055, 0.77 ± 0.047, and 0.70 ± 0.092. Lineage distribution models indicated that each lineage occurred over different geographical areas with more or less contact areas at margins (Figure 4). The suitability map reconstructed based only on the occurrences of three aberrant *Ajania* species of Korea-Japan distribution was congruent with those of other *Ch. indicum*-complex species (Figures 2, 4 and Supplementary Figure 5D).

**FIGURE 5:**
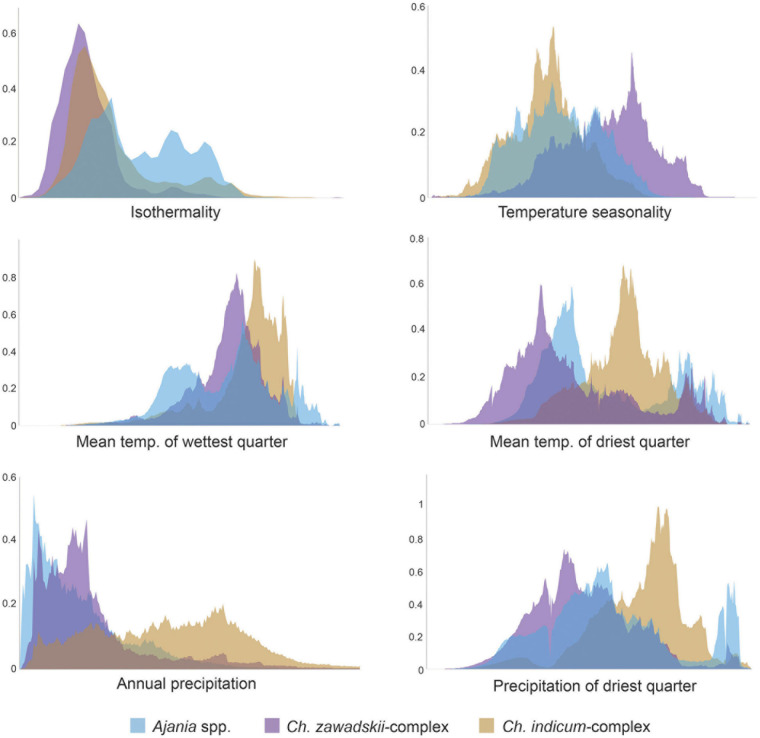
Diagrams displaying niche overlaps among three major lineages. Suitability diagrams were converted from potential distribution and ecological data according to the strategy reported by Evans et al. (2009). The *x*-axis indicates the adaptive range of such ecological factors, and the *y*-axis indicates suitability. Different colors correspond to different lineages, as shown in the legend at the bottom.

Considering the predicted distribution of the modeled lineages (Figure 4), the comprehensive pairwise *D*-values among lineages ranged from 0.34 to 0.4 (Table 5), suggesting ecological differentiation (Figure 4 and Table 5). The background niche similarity tests indicated that the observed overlaps were greater than 95% of the simulated values, suggesting that lineages occupy an ecological space that is more similar to each other than expected by chance (Table 5). However, the null hypothesis of niche equivalency was rejected for all comparisons between subclades. The profiling niche occupancy (PNO) of each ecological factor was computed for the detailed adaptation patterns (Supplementary Table 4). As a result, *Ajania* had the lowest niche overlap with two subclades of *Chrysanthemum* in precipitation in the driest quarter and isothermality (*D*-values of 0.4 and 0.46) (Figure 5 and Supplementary Table 4). Two *Chrysanthemum* subclades greatly diverged in temperature seasonality and mean temperature in the driest quarter (Figure 5 and Supplementary Table 4, *D*-value of ca. 0.46).

**TABLE 5:** Ecological niche comparison among three subclades of the *Chrysanthemum*-group.

**Comparisons**		**Niche overlap**	**Niche overlap**	**Niche similarity**		**Niche**
A	b	(*D*-index)	(*I*-index)	a vs. b	b vs. a	**equivalency**
Aj	Ci	0.3389	0.5312	Similar**	Similar**	Different**
Aj	Cz	0.3948	0.5827	Similar**	Similar**	Different**
Ci	Cz	0.4101	0.6017	Similar**	Similar**	Different**

Pairwise niche overlap values are presented for the comparison of niche similarity and equivalency of subclade a with subclade b in terms of Schoener’s *D* and modified Hellinger distances (*I*-values). Aj, *Ajania*; Ci, *Ch. indicum*-complex; Cz, *Ch. zawadskii*-complex. Niche similarity *P*-values and equivalency via randomization test: **The comprehensive niche is significantly (*P* < 0.01) more similar or different than expected due to randomness. Pairwise niche overlap values for each essential niche are also shown.

## Discussion

### Clarifying Phylogenetic Positions of Some Taxa in Subtribe Artemisiinae

Despite a series of cladistical studies based on morphological and molecular information, there remain quite a number of taxa whose systematic positions are unclear in subtribe Artemisiinae (Bremer and Humphries, 1993; Torrell et al., 1999; Martin et al., 2001; Vallès and McArthur, 2001; Vallès et al., 2003; Oberprieler et al., 2007; Sanz et al., 2008; Zhao et al., 2010a, b). *Phaeostima* is one such taxon. It was separated from *Ajania* as an independent genus by Muldashev (1981). However, neither morphological data nor molecular phylogenies could resolve its relationships with *Ajania*, *Artemisia*, and *Chrysanthemum* (see Huang et al., 2017 and references therein). By integrating morphological analyses of 20 characters, and palynological and molecular data, Huang et al. (2017) circumscribed a monophyletic *Phaeostigma* by including three species formerly named under *Ajania*, which was in line with the proposal of Bremer and Humphries (1993) that the circumscription of *Phaeostigma* should be extended to some members of *Ajania*. Moreover, Huang et al. (2017) supposed that *Phaeostigma* might be phylogenetically closer to *Artemisia* than to *Ajania* (Huang et al., 2017; Chen et al., 2020), which was strongly supported by the present analysis, that is, *Phaeostigma* was nested in the clade of *Artemisia*-group (Figure 1). Nevertheless, the similarity between *Ajania* and *Phaeostigma* in terms of characteristics such as synflorescence and capitulum, as well as in diversification ages and distribution patterns, implies that they may have experienced convergent evolution under similar habitats (Figures 1, 2 and Supplementary Figure 2). More detailed studies are required to determine the adaptive significance of these traits.

*Opisthopappus* is another taxon worth discussion. It was placed in subtribe Tanacetinae by Bremer and Humphries (1993) mainly due to the presence of pappi or coronas on achenes but was different from other members of Tanacetinae according to its myxogenic achenes. Its systematic position in subtribe Tanacetinae was called into question by the present (Figure 2) and previous (Zhao et al., 2010a, b) molecular data that suggested a closer relationship to *Chrysanthemum* and *Ajania*. Besides, several studies reported crossability between *Opisthopappus* and species of *Chrysanthemum*-group, e.g., *Ch. lavandulifolium*, *Ch. dichrum*, *A. pallasiana*, and *Elachanthemum intricatum* and *A. pacifica* x *Ch. vestitum* (Hu and Zhao, 2008; Yang et al., 2010; Zheng et al., 2013). Combining all the evidence, we suggest a taxonomic treatment to incorporate *Opisthopappus* into *Chrysanthemum*-group. Nevertheless, to clarify its phylogenetic position, more data of morphological and ecological traits, reproductive biology and phylogenomics are required.

The systematic positions of *Hippolytia*, *Nipponanthemum*, *Leucanthemella*, *Brachanthemum*, and *Tanacetum tatsienense* have also been the subjects of much debate. *Hippolytia* and *Tanacetum* were formerly described in subtribe Tanacetinae, while *Nipponanthemum* and *Leucanthemella* were classified into subtribe Leucantheminae, but all were later shown to belong to subtribe Artemisiinae according to molecular phylogenetic data (Vallès and McArthur, 2001; Watson et al., 2002; Vallès et al., 2003; Oberprieler, 2005; Oberprieler et al., 2007, 2009; Masuda et al., 2009). Using multiple nuclear loci with a coalescent analytical method, we found that they belonged to Artemisiinae, which was supported by our analysis. Meanwhile, our analysis showed that *Hippolytia*, *Nipponanthemum*, *Leucanthemella*, *Tanacetum tatsienense*, and *Brachanthemum* were nested into different branches of *Artemisia*-group (Figure 1) rather than being basal grades of Artemisiinae, as suggested by previous ITS-ETS data (Watson et al., 2002; Sanz et al., 2008; Zhao et al., 2010a).

### Evolution of the Capitulum Architecture in Subtribe Artemisiinae

Subtribe Artemsiinae is one of the youngest lineages in the daisy family, and it appears as an assemblage of taxa of plesiomorphic features, especially with regard to pollination syndrome, including capitulum architectures and affiliated features (Harris, 1999; Martin et al., 2001; Sanz et al., 2008; Pellicer et al., 2010; Huang et al., 2017). In terms of capitulum architecture, three types can be observed in wild Artemisiinae species: radiate, disciform and discoid. The radiate capitula are composed of many central disk florets and peripheral conspicuous ray flowers, whereas, the discoid consist of bisexual disk flowers only, and the disciform are discoid-like but with a few tubular female marginal florets). The evolutionary orientation of these capitulum types has remained a key question in teasing apart historical patterns of lineage divergence and/or hybridization within Artemisiinae. Bremer and Humphries (1993) regarded the radiate capitulum as the plesiomorphy of Artemisiinae and the discoid as a derivative of the disciform, rather than evolving directly from the radiate. However, ancestral state reconstruction based on the ITS-ETS tree suggested that the discoid was the plesiomorphy of Artemisiinae (Sanz et al., 2008). In the present study, using a similar strategy, we tried to detect the direction of capitulum evolution in Artemisiinae. The results also showed discoid or disciform flowerheads as ancestral states (Supplementary Figure 6). However, model-based ancestral character reconstruction of a particular trait may not provide really accurate information without mechanism evidences, because missing data of extinct taxa or bias in models for state transition might influence the accuracy of the inference (Omland, 1999; Griffith et al., 2015). A clear answer to the problem should rely on evidence from evolutionary developmental genetic studies. Trow (1912), as well as Ford and Gottlieb (1990) examined the inheritance of rayed and rayless heads in *Senecio* and *Layia*, respectively, and suggested that the switch between the presence and absence of ray flowers may be governed by simple genetic rules. Later molecular genetic studies indicated that the CYC2-mediated module participates in inflorescence repatterning (Broholm et al., 2008; Chapman et al., 2008, 2012; Kim et al., 2008; Tähtiharju et al., 2012; Chen et al., 2018), and the rise of rayless capitula is always linked to a reduction in the expression or gene loss of one or more CYC2 members (Chen et al., 2018). Our recent analyses demonstrated that dysfunction of *CYC2g* led to a shift from the radiate flowerhead to the disciform head in *Chrysanthemum*-group (Shen et al., 2021). Therefore, the radiate should be the ancestral state of the capitulum in subtribe Artemisiinae, despite *Stilpnolepis centiflora* at its basal position possessing discoid flowerheads (probably an autoapomorphic state). The shifts from radiate to disciform capitula may have happened repeatedly during the diversification of this group. Early on, in 1961, Tzvelev considered that *Ajania* and *Artemisia* were convergent lineages evolved independently from *Chrysanthemum*-like ancestors. Similarly, our phylogenetic tree suggested that convergent capitula evolution may also have occurred within the *Artemisia*-group (Figure 1). For example, the ‘disciform’ capitula in *Artemisia* look similar with, but may have different developmental underpinnings from that of *Ajania*. Therefore, the contradiction between the reconstructed ancestral state of capitulum of subtribe Artemisiinae (Sanz et al., 2008) and the evo-devo insights is probably due to the morphological convergence.

To date, there have been no data demonstrating whether there is a transition from the discoid to the disciform capitula. During our previous morphogenetic study (Ren and Guo, 2015), a uniformly acropetal developmental sequence was found on the homogamous discoid capitulum, but basipetal development probably occurred in the marginal florets of the heterogamous radiate/disciform flowerhead. This was also postulated by Harris (1995). Although functional assays of chrysanthemum *ClCYC2g* and sunflower *HaCYC2c* showed that downregulating the expression of these genes led to the appearance of dorsal petals and stamens in ray florets, we assume that the developmental process of the discoid capitulum might be completely different from that of the radiate/disciform due to lacking a module responsible for the development of zygomorphic or asymmetric marginal female flowers, namely, a marginality identity module (Pozner et al., 2012; Zhao et al., 2016; Elomaa et al., 2018; Zoulias et al., 2019; Shen et al., 2021). The finding of our previous study that the *CYC2g* gene was lost in the discoid species *Stilpnolepis centiflora* (Chen et al., 2018; Shen et al., 2021) may be a hint for this hypothesis. Certainly, to test this hypothesis, more detailed molecular developmental studies are required.

### Flowerhead Morphology Associated With Climatic Niche Divergence as a Driver of Species Diversification of *Chrysanthemum*-Group

As mentioned above, the relationship between *Chrysanthemum* and *Ajania* and their patterns of diversification and evolution have remained highly controversial. Considering their distinct capitulum morphology and geographic distribution, with members of *Ajania* being more western than *Chrysanthemum*, and members of *Ch. indicum*-complex being more southeastern than *Ch. zawadskii*-complex (Figure 4 and Supplementary Figure 5), we postulate that the three main taxa must have experienced morphological differentiation under niche divergence.

From the environmental aspect, different landscape features and climate patterns can drive directional selection (Sobel et al., 2010; Rundle and Nosil, 2012). According to ^∗^BEAST analysis, separation between *Chrysanthemum*-group and *Artemisia*-group occurred around the late Miocene (ca. 9 Ma, Figure 1), and the radiation within *Chrysanthemum*-group was dated to ca. 2 Ma. The time frame largely corresponded to the period of the profound climatic change in Asia interior, i.e., extremely intensive aridification in Middle to Central Asia demonstrated by paleoenvironmental data on loess and clay deposits (An et al., 2001; Ma et al., 2005; Nie et al., 2014; Wang X. et al., 2016), which may be due to the synergy of recently geological activity of Northern QTP (Miao et al., 2012; Favre et al., 2015; Shi et al., 2019; Yang et al., 2019). Our DIVA analysis also indicated the origin of *Chrysanthemum*-group in Central Asia, followed by an eastward dispersal of the *Chrysanthemum* lineage, and *in situ* diversification or colonization of the *Ajania* lineage along with tectonic activities of QTP (Table 3 and Supplementary Figure 2; Chen et al., 2020). Thus, the late Miocene origin and evolution of *Chrysanthemum*-group was strongly influenced by the establishment and development of climatic heterogeneity in Central-East Asia.

In Asteraceae, alterations of capitulum forms are closely associated with shifts in reproductive strategies, and therefore, variations in capitulum architecture should be consequences of trade-offs between energy consumption and adaptation in response to environmental stresses (Stuessy et al., 1986; Berry and Calvo, 1989; Andersson, 1999; Nielsen et al., 2002; Chen et al., 2020). Our previous analyses have demonstrated that the radiate flowerhead with white ligules (of *Chrysanthemum zawadskii-*complex) represents an earlier evolutionary status, and the disciform flowerhead of *Ajania* evolved from the radiate at approximately 8 Ma, somewhat overlapping with the time when *Chrysanthemum*-group arose but occurring much earlier than the divergence time of *Chrysanthemum* and *Ajania* (Figure 1 and Supplementary Figure 7). This suggests that capitulum polymorphism had long existed in the ancestral populations of the *Chrysanthemum-*group. Comparing the present multilocus coalescent tree and the tree based on *CYC2g* sequences of *Chrysanthemum*-group (Shen et al., 2021), we found a high consensus of topologies, implying that the evolution of this group is linked to the alteration of flowerhead morphology (Supplementary Figure 7). Within *Chrysanthemum*-group, low pairwise niche overlaps were found between subclades, although they diverged rather recently (Figures 4, 5). The ecological suitability diagrams based on the current climate scenario suggested that the *Ajania* lineage preferred environments with lower yearly mean temperatures and precipitation levels, together with higher climatic instability, compared to the two *Chrysanthemum* lineages, which is consistent with the habitat shift between the plants being related to anemophily and entomophily (Culley et al., 2002) and is concordant with the good differentiation of their capitulum morphologies (Figure 5).

Shown in Figure 2, three Korea-Japan distributed *Ajania* species, *A. pallasiana_JJ*, *A. pacifica*, and *A. shiwogiku*, fell into the clade of *Ch. indicum*-complex. This could be due to incomplete lineage sorting of genes analyzed here. We found that the sequences of their *CYC2g* genes were highly similar to those of *A. parviflora* (Shen et al., 2021; Supplementary Figure 7) which is a member distributed in the eastern part of the range of *Ajania*. However, the mix-up of three *Ajania* species with *Chrysanthemum* is more likely due to secondary contacts as we found all three species are polyploids with high ploidy levels from 2n = 6x to 8x and to 10x, suggesting complicated origins probably involving parentages from *Ch. indicum*-complex. The present SDM analysis suggested a broader geological overlap between *Ajania* and *Chrysanthemum* during the LGM (Figure 4), implying possible introgression between the two recently diverging lineages because morphological differentiation and reproductive barriers between them might still be incomplete (Comes and Kadereit, 1998; Potts et al., 2003; Hewitt, 2004; Li et al., 2013; Chen et al., 2020).

Overall, considering the divergence time, ancestral distribution, and niche differentiation data, we propose that the divergence of three major groups in *Chrysanthemum-*group, *Ajania*, *Ch. indicum*-complex, and *Ch. zawadskii*-complex, probably occurred in a rather short time span in response to environmental heterogeneity in interior East Asia, which might be related to divergence of capitulum types.

## Data Availability Statement

The datasets presented in this study can be found in online repositories. The names of the repository/repositories and accession number(s) can be found in the article/Supplementary Material.

## Author Contributions

C-ZS conceived the study, performed the most of the experiments, and data analyses. C-JZ and JC participated in the experiments. C-ZS and Y-PG wrote the manuscript. All the authors have read and approved the manuscript.

## Conflict of Interest

The authors declare that the research was conducted in the absence of any commercial or financial relationships that could be construed as a potential conflict of interest.

## Footnotes

1 www.jelitto.com

2 https://mafft.cbrc.jp/alignment/server/

3 http://www.phylo.org/

4 https://www.worldclim.org

5 https://www.gbif.org

6 http://www.nsii.org.cn/2017/home.php

## Supplementary Material

Supplementary Figure 1 Bayesian trees of the single-copy nuclear genes.

Supplementary Figure 2 Historical biogeographical analyses of subtribe Artemisiinae.

Supplementary Figure 3 Time frame comparison among four different calibration settings.

Supplementary Figure 4 Principal component analysis (PCA) of environmental factors of the *Chrysanthemum*-group.

Supplementary Figure 5 Potential suitability distribution of climate conditions of major lineages within the *Chrysanthemum*-group.

Supplementary Figure 6 Ancestral state reconstruction of capitulum architectures based on the phylogenetic framework under likelihood model **(right)** and continuous-time Markov model **(left)**.

Supplementary Figure 7 Comparison of the gene tree of *CYC2g* that regulates capitulum architectures in subtribe Artemisiinae and the present multilocus phylogeny (left, redrawn from Figures 1, 2 in the main text).

Supplementary Table 1 Species used in the present study and the sampling information.

Supplementary Table 2 GPS coordinates used for niche modeling in this study.

Supplementary Table 3 Biogeographic history of subtribe Artemisiinae reconstructed with DEC and s-DEC.

Supplementary Table 4 Pairwise niche overlap among three subclades of the *Chrysanthemum*-group under predicted niche occupancy profiles (PNO).

Supplementary Data Sheet 1 Datasets of the sequence alignments.
