# Supplementary figures and images for: Clarifying Recent Adaptive Diversification of the Chrysanthemum-Group on the Basis of an Updated Multilocus Phylogeny of Subtribe Artemisiinae (Asteraceae: Anthemideae)
Source: Front Plant Sci. 2021 May 26;12:648026. doi: 10.3389/fpls.2021.648026 (PMC8187803; doi:10.3389/fpls.2021.648026)

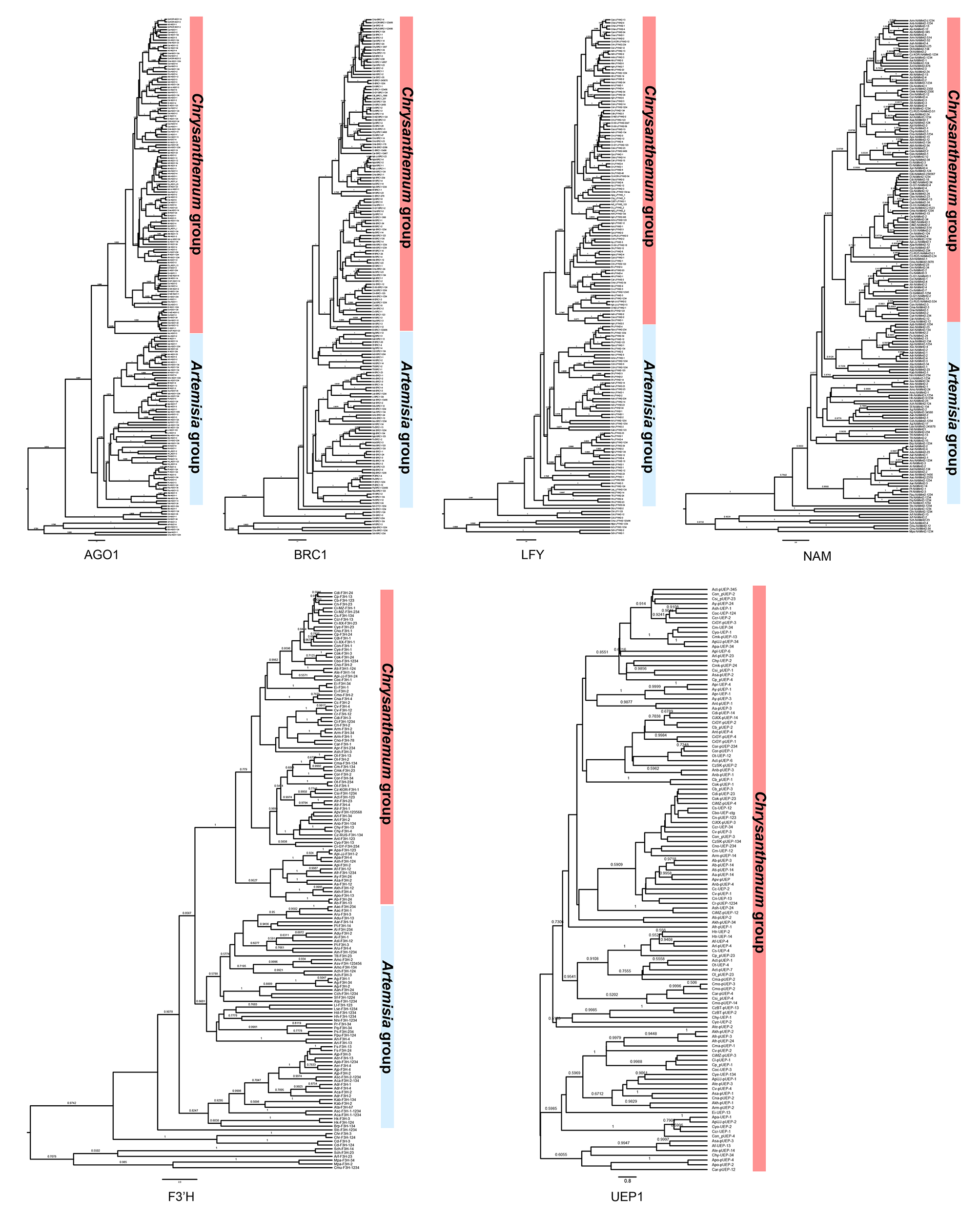

Supplement: Supplementary Figure 1 — Bayesian trees of the single-copy nuclear genes. [file Image_1.PNG]

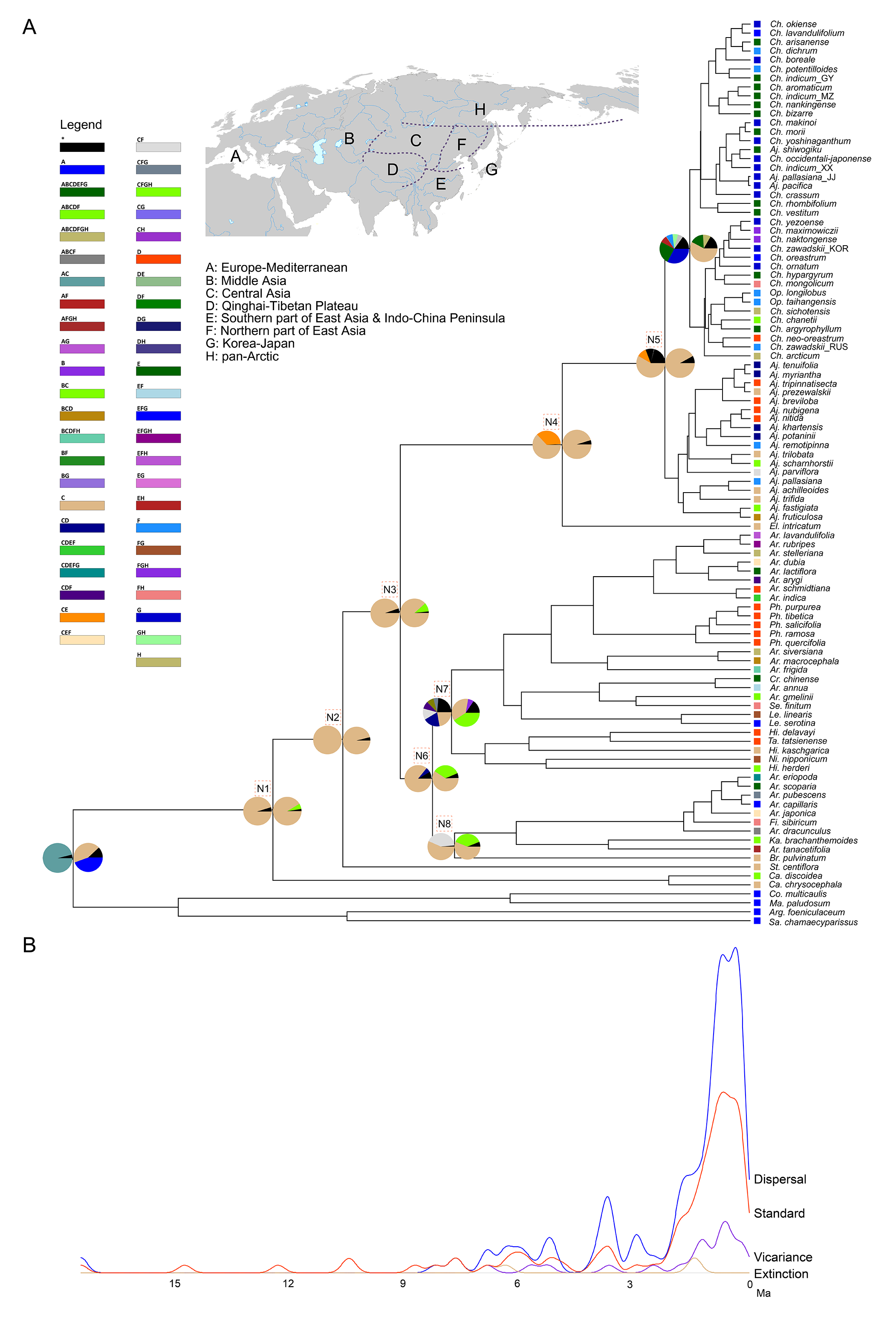

Supplement: Supplementary Figure 2 — Historical biogeographical analyses of subtribe Artemisiinae. [file Image_2.PNG]

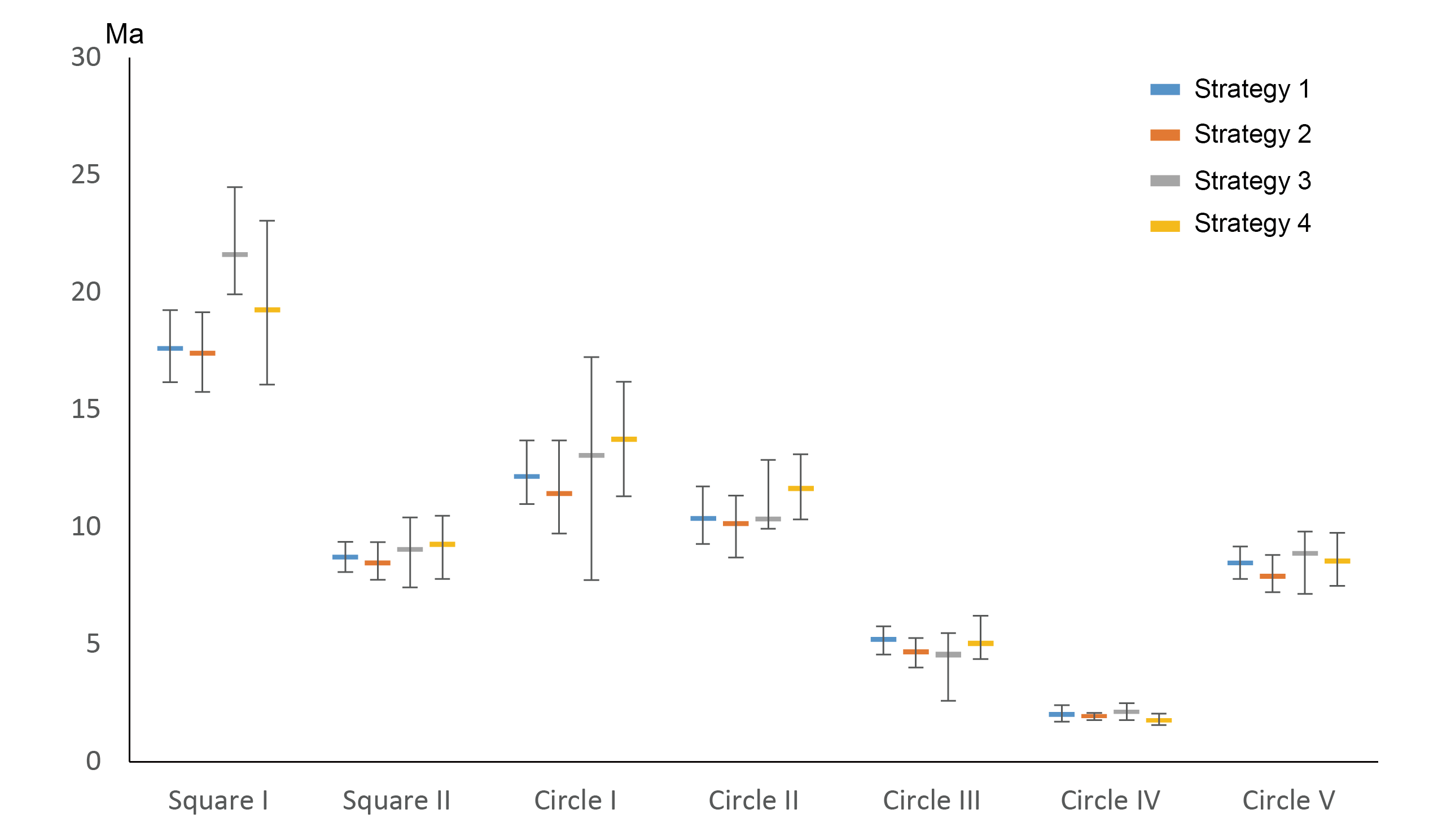

Supplement: Supplementary Figure 3 — Time frame comparison among four different calibration settings. [file Image_3.PNG]

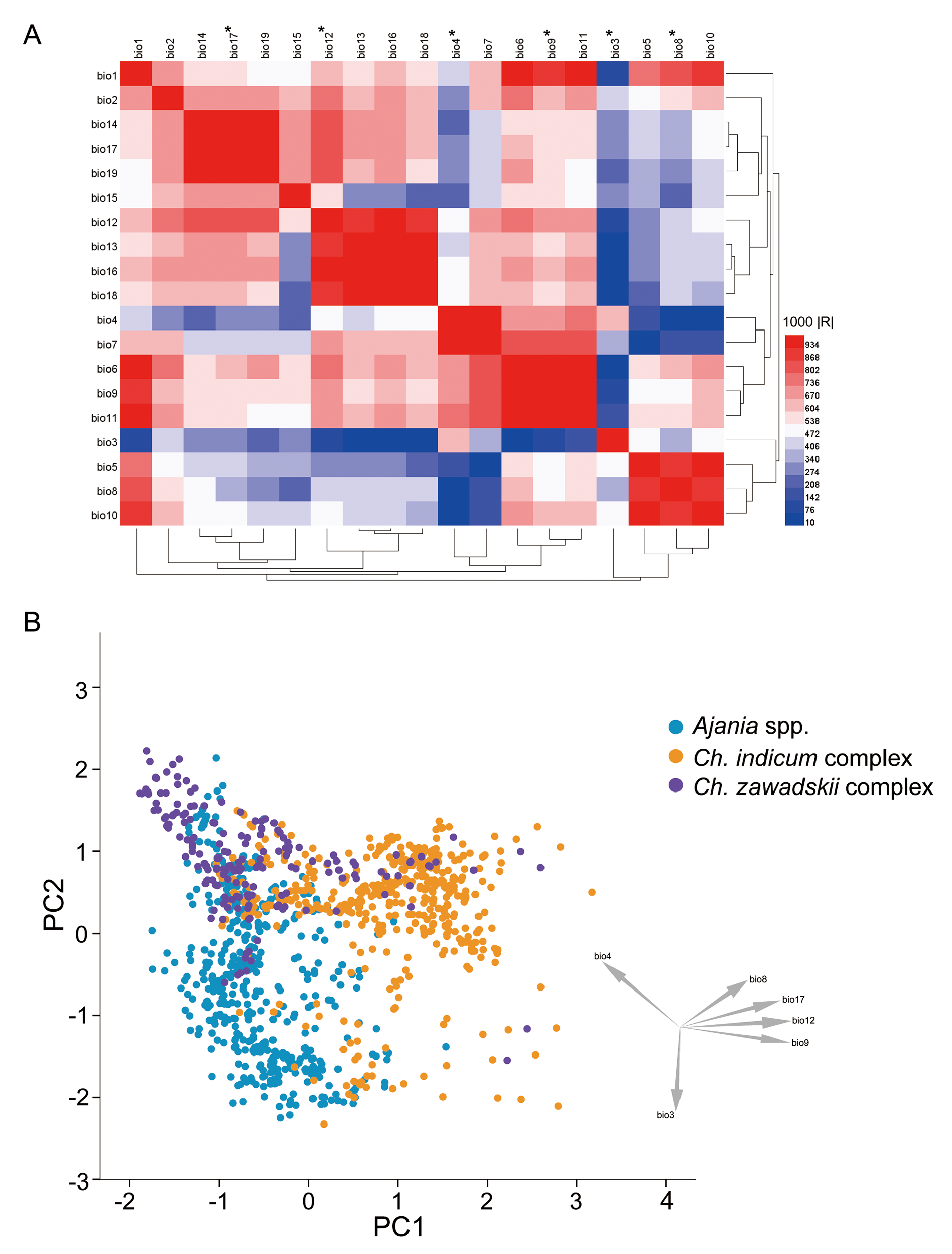

Supplement: Supplementary Figure 4 — Principal component analysis (PCA) of environmental factors of the Chrysanthemum-group. [file Image_4.PNG]

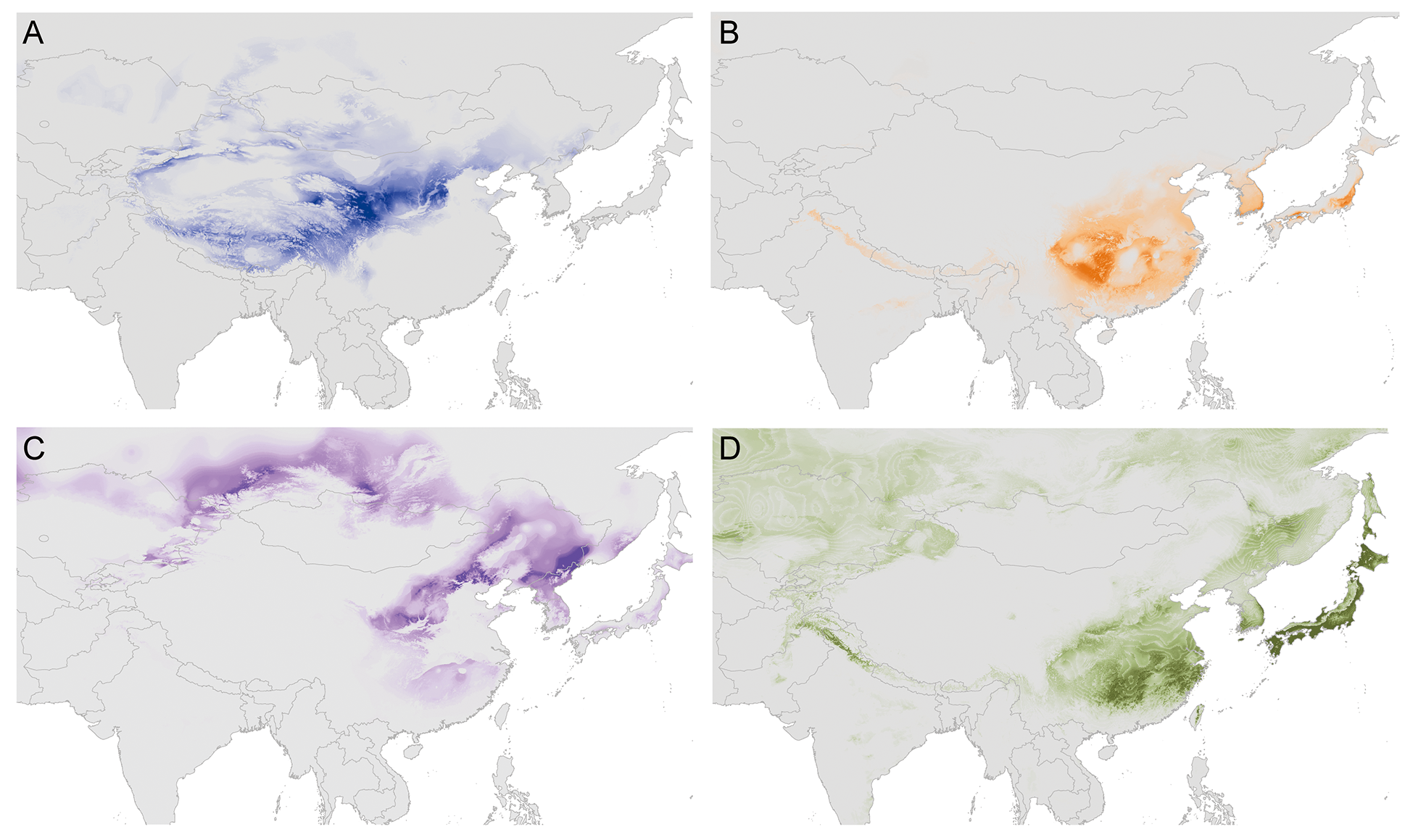

Supplement: Supplementary Figure 5 — Potential suitability distribution of climate conditions of major lineages within the Chrysanthemum-group. [file Image_5.PNG]

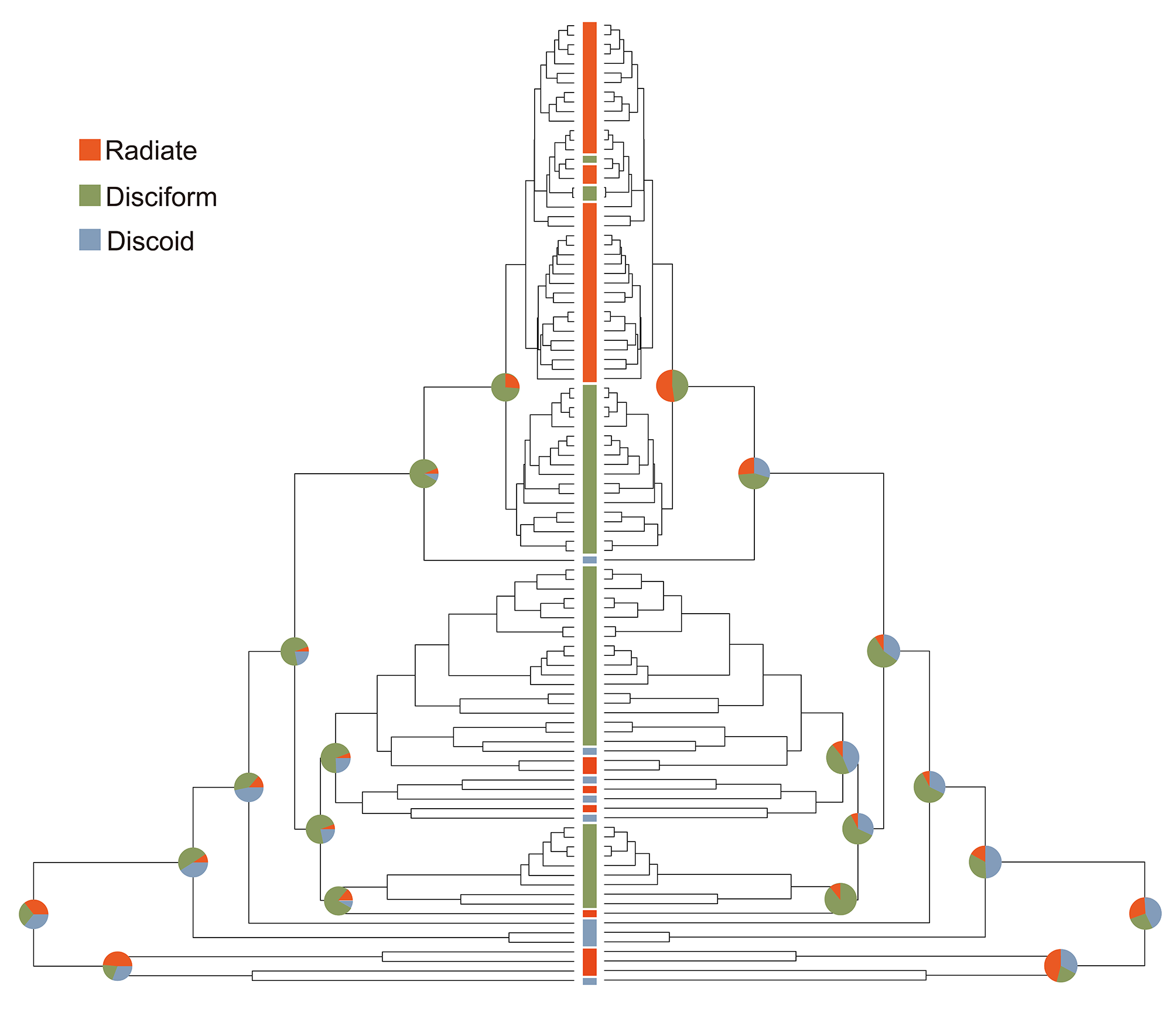

Supplement: Supplementary Figure 6 — Ancestral state reconstruction of capitulum architectures based on the phylogenetic framework under likelihood model (right) and continuous-time Markov model (left). [file Image_6.PNG]

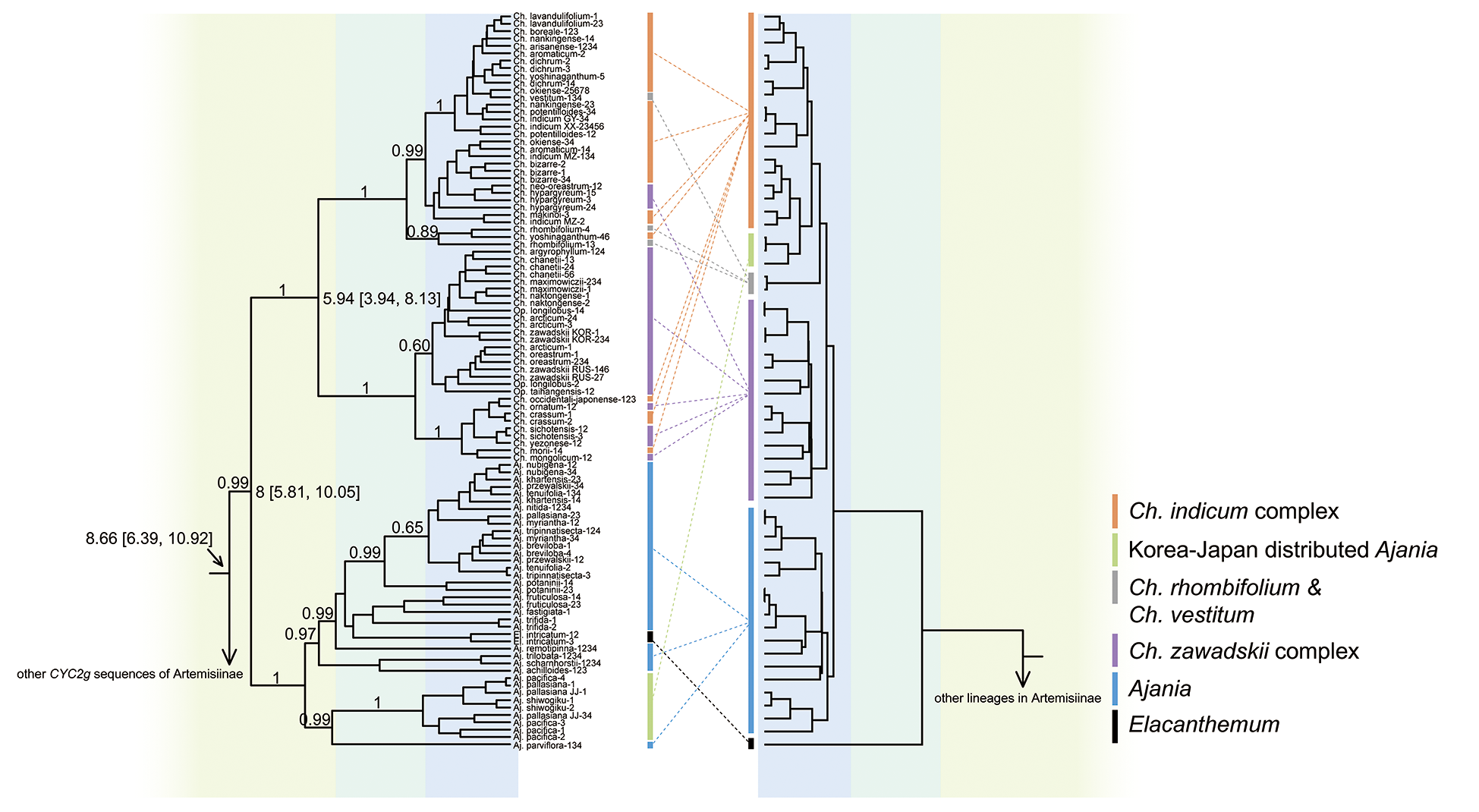

Supplement: Supplementary Figure 7 — Comparison of the gene tree of CYC2g that regulates capitulum architectures in subtribe Artemisiinae and the present multilocus phylogeny (left, redrawn from Figures 1, 2 in the main text). [file Image_7.PNG]
