# Supplementary material for: Clarifying Recent Adaptive Diversification of the Chrysanthemum-Group on the Basis of an Updated Multilocus Phylogeny of Subtribe Artemisiinae (Asteraceae: Anthemideae)
Source: Front Plant Sci. 2021 May 26;12:648026. doi: 10.3389/fpls.2021.648026 (PMC8187803; doi:10.3389/fpls.2021.648026)
Supplement: Supplementary file 13 [file Data_Sheet_2.docx]

**Figure. S1.** Bayesian trees of the single-copy nuclear genes. The tree of *UEP1* only contains samples of the *Chrysanthemum*-group as it could not be amplified from some of the *Artemisia* and the outgroup species. These trees were constructed in BEAST under their best-fit evolutionary models as shown in Table 2, and by selecting the Yule prior. Bayesian inference was conducted every 10000 generations for a total of 400 million generations. Every tree was exported with discarding the first 30% states as burn-in. Posterior probabilities (>0.5) are indicated above branches. All haplotype sequences from each of the sampled accessions are shown on the terminal branches which are coded by abbreviation of species names plus haplotype codes. Abbreviations of species names are shown in Table S1.

**Figure S2.** Historical biogeographical analyses of subtribe Artemisiinae. A. Phylogenetic tree with ancestral distribution ranges estimated using s-DIVA and BBM models. Eight geographical units (A–G) across the Eurasia were defined according to the overall distribution of sampled species and their geographical features. Current geographic range for each taxon is marked in different colors with area name attached to the terminal node. Pie charts at major internal nodes represent marginal probabilities of possible ancestral areas (of each pie chart, the left from s-DIVA and the right from BBM; black color represents uncertainty). B. Time-Event curves showing historical dynamics of biogeography of subtribe Artemisiinae. Lines in grey, carnation, green and black represent dynamics of dispersal, vicariance, standard and extinction, respectively.

**Figure S3.** Time frame comparison among four different calibration settings. The node ages and their 95% confidence intervals of major nodes were shown with different colors. Strategy 1: two secondary calibration points were set, which corresponded to the divergence time of the Eurasia-Mediterranean grade and the Asian-southern African grade, as well as the age of the crown clade of Artemisiinae according to Oberprieler (2005). Strategy 2: one secondary calibration point corresponding to the divergence time of Artemisiinae and Santolininae (Tomasello et al., 2015). Strategy 3: one secondary calibration point was set corresponding to the divergence time of Artemisiinae-Santolininae-Glebionidinae and other Euroaisa lineages (Tomasello et al., 2015). Strategy 4: one secondary calibration point set at the age of crown clade of Artemisiinae based on Artemisia-like pollen fossils.

**Figure S4.** Principal component analysis (PCA) of environmental factors of the *Chrysanthemum* group. A. Heatmap displaying the correlation coefficient of each pair of ecological factors. Colors indicates the absolute value of R x 1000. The clustering analysis that indicating the correlation of these factors was conducted based on pairwise correlation coefficients. B. PCA scatter plot generated from six less correlated ecological factors (bio varieties signed with “*”). Blue, yellow and purple spots indicate niches of species of *Ajania*, *Ch. indicum* complex and *Ch. zawadskii* complex, respectively. Grey arrows draw the weights of climatic factors according to the information shown in Table 4 in the main text. Abbreviations: bio1, annual mean temperature; bio2, mean diurnal range; bio3, isothermality; bio4, temperature seasonality; bio5, max temperature of warmest month; bio6, min temperature of coldest month; bio7, temperature annual range; bio8, mean temperature of wettest quarter; bio9, mean temperature of driest quarter; bio10, mean temperature of warmest quarter; bio11, mean temperature of coldest quarter; bio12, annual precipitation; bio13, precipitation of wettest month; bio14, precipitation of driest month; bio15, precipitation seasonality; bio16, precipitation of wettest quarter; bio17, precipitation of driest quarter; bio18, precipitation of wettest month; bio19, precipitation of driest month.

**Figure S5.** Potential suitability distribution of climate conditions of major lineages within *Chrysanthemum* group. A, B and C show the estimated suitability distributions of *Ajania*, *Ch. indicum* complex, *Ch. zawadskii* complex, respectively, under DIVA-GIS during the current climate scenario. D. SDM of the Korea-Japan distributed *Ajania* species under MAXENT, showing similar suitability pattern with *Ch. indicum* complex (also see Figure 4 in the main text). Shading in color represents the probability/suitability for each subclade.

**Figure S6.** Ancestral state reconstruction of capitulum architectures based on the phylogenetic framework under likelihood model (right) and continuous-time Markov model (left). Colors representing different character states are marked on the terminal nodes as the legend in upper-left. Pie charts at major internal nodes showing probabilities of each possible ancestral state.

**Figure S7.** Comparison of the gene tree of *CYC2g* that regulates capitulum architectures in Subtribe Artemisiinae and the present multilocus phylogeny (Left, redrawn from the Figures 1 & 2 in the main text). The construction of *CYC2g* gene tree was based on data of Shen et al. unpubl. with Bayesian posterior probabilities higher than 0.5 indicated on branches, and divergence times and their 95% HPDs of major clades. Bars in different colors mean different lineages. Blocks in blue, green and yellow represent Quaternary, Pliocene and Miocene respectively.
